# Supplementary material for: Modelling Skylarks (Alauda arvensis) to Predict Impacts of Changes in Land Management and Policy: Development and Testing of an Agent-Based Model
Source: PLoS One. 2013 Jun 6;8(6):e65803. doi: 10.1371/journal.pone.0065803 (PMC3675089; doi:10.1371/journal.pone.0065803)
Supplement: Supporting Information S4 — The skylark ODdox as a zipped archive. (ZIP) [file pone.0065803.s004.zip › Skylark_ODdox/skylarks__all_8cpp.html]

ALMaSS Skylark ODdox: skylarks\_all.cpp File Reference


ALMaSS Skylark ODdox
 2.0


- Main Page
- Related Pages
- Classes
- Files

- File List
- File Members

Macros |
Functions |
Variables

skylarks\_all.cpp File Reference

`#include <string.h>`  
`#include <cmath>`  
`#include <vector>`  
`#include <algorithm>`  
`#include <iostream>`  
`#include <fstream>`  
`#include "../ALMaSSDefines.h"`  
`#include "../Landscape/ls.h"`  
`#include "../BatchALMaSS/populationmanager.h"`  
`#include "../Skylark/skylarks_all.h"`  
`#include "../BatchALMaSS/BoostRandomGenerators.h"`

|  |  |
| --- | --- |
| Macros | |
| #define | \_\_CJTDebug\_7 |
| #define | SKOPTIMALHABITATSCORE   50 |

|  |  |
| --- | --- |
| Functions | |
| bool | CompareDist (Point i, Point j) |

|  |  |
| --- | --- |
| Variables | |
| static int | Breed\_Res\_Thresh1 |
| const double | Breed\_Res\_Thresh2 = Breed\_Res\_Thresh1 \* 4 |
| const double | Breed\_Temp\_Thresh = 5.0 |
| const double | CE\_nest [15] |
| CfgInt | cfg\_adultreturnmort |
|  | Immigration mortality for juveniles. |
| CfgInt | cfg\_Breed\_Res\_Thresh1 |
| CfgBool | cfg\_CIPEGridOutput\_used |
| CfgInt | cfg\_ClutchMortProb |
| CfgFloat | cfg\_ConversionEffReduc |
| CfgFloat | cfg\_Cooling\_Rate\_Eggs |
|  | Maximum immigration mortality for females. |
| CfgFloat | cfg\_densityconstant\_a |
| CfgFloat | cfg\_densityconstant\_b |
| CfgInt | cfg\_densityconstant\_c |
| CfgFloat | cfg\_EggTemp |
| CfgFloat | cfg\_EM\_Nestling\_a |
| CfgFloat | cfg\_EM\_Nestling\_b |
| CfgInt | cfg\_fecundity\_reduc |
| CfgInt | cfg\_fecundity\_reduc\_chance |
| CfgFloat | cfg\_FemaleMinTerritoryAcceptScore |
| CfgInt | cfg\_FoodTripsPerDay |
| CfgFloat | cfg\_heightconstant\_a |
| CfgFloat | cfg\_heightconstant\_b |
| CfgInt | cfg\_heightconstant\_c |
| CfgFloat | cfg\_hindconstantD\_b |
| CfgFloat | cfg\_hindconstantH\_b |
| CfgInt | cfg\_HQualityBareEarth |
| CfgInt | cfg\_HQualityHedge |
| CfgInt | cfg\_HQualityHedgeScrub |
| CfgInt | cfg\_HQualityMetalRoad |
| CfgInt | cfg\_HQualityNeutral |
| CfgInt | cfg\_HQualityOpenTallVeg |
| CfgInt | cfg\_HQualityTall |
| CfgInt | cfg\_HQualityTall2 |
| CfgInt | cfg\_HQualityTallVeg |
| CfgInt | cfg\_HQualityTrack |
| CfgInt | cfg\_HQualityVeg30cm |
| CfgInt | cfg\_HQualityWater |
| CfgInt | cfg\_insecticide\_direct\_mortE |
| CfgInt | cfg\_insecticide\_direct\_mortF |
| CfgInt | cfg\_insecticide\_direct\_mortM |
| CfgInt | cfg\_insecticide\_direct\_mortN |
| CfgInt | cfg\_insecticide\_direct\_mortP |
| CfgInt | cfg\_juvreturnmort |
|  | Immigration mortality for juveniles. |
| CfgFloat | cfg\_MaleSplitScale |
| CfgFloat | cfg\_maxfeedrain |
| CfgFloat | cfg\_MD\_Threshold |
| CfgFloat | cfg\_MeanExtractionRatePerMinute |
| CfgFloat | cfg\_MeanHatchingWeight |
| CfgFloat | cfg\_MinDaysToHatch |
|  | The time taken to egg hatch under optimal conditions. |
| CfgInt | cfg\_NestLeavingChance |
| CfgFloat | cfg\_NestLeavingWeight |
| CfgInt | cfg\_NestlingMortProb |
| CfgFloat | cfg\_NestPlacementMinQual |
| CfgInt | cfg\_PatchyPremium |
| CfgFloat | cfg\_PEmax |
| CfgInt | cfg\_pm\_eventfrequency |
| CfgInt | cfg\_pm\_eventsize |
| CfgInt | cfg\_PreFledgeMortProb |
| CfgInt | cfg\_rainhindpow |
| CfgBool | cfg\_ReallyBigOutput\_used |
| CfgBool | cfg\_ReallyBigOutputMonthly\_used |
| CfgBool | cfg\_RipleysOutput\_used |
| CfgFloat | cfg\_SK\_hindrance\_scale |
| CfgFloat | cfg\_sk\_triplength |
| CfgFloat | cfg\_SkScrapesPremiumII |
| CfgInt | cfg\_SkStartNos |
|  | The number of skylarks that start in the simulation. |
| CfgInt | cfg\_SkTramlinesPremium |
| CfgFloat | cfg\_Skylark\_female\_Biodegredation |
|  | The proportion of pesticide remaining from one day to the next for females. |
| CfgFloat | cfg\_Skylark\_female\_NOEL |
|  | Can be used to trigger a response to pesticides for the females. |
| CfgFloat | cfg\_Skylark\_male\_Biodegredation |
|  | The proportion of pesticide remaining from one day to the next for males. |
| CfgFloat | cfg\_Skylark\_male\_NOEL |
|  | Can be used to trigger a response to pesticides for the males. |
| CfgFloat | cfg\_Skylark\_nestling\_Biodegredation |
|  | The proportion of pesticide accumulated from one day to the next. |
| CfgFloat | cfg\_Skylark\_nestling\_NOEL |
|  | Can be used to trigger a response to pesticides for the nestlings. |
| CfgFloat | cfg\_Skylark\_prefledegling\_Biodegredation |
|  | The proportion of pesticide remaining from one day to the next for prefledglings. |
| CfgFloat | cfg\_Skylark\_prefledegling\_NOEL |
|  | Used for determining the pesticide response for prefledglings. |
| CfgInt | cfg\_strigling\_clutch |
| CfgInt | cfg\_strigling\_nestling |
| CfgInt | cfg\_strigling\_preflg |
| CfgInt | cfg\_temphindpow |
| CfgFloat | cfg\_tramline\_foraging |
| static int | ClutchMortProb |
| static double | Cooling\_Rate\_Eggs |
| double | DensityScore [111] |
| static double | EggTemp |
| const double | EM\_coldblood1 = 0.4257 |
| const double | EM\_coldblood2 = 0.482 |
| const double | EM\_nest\_T [14] |
| const double | EM\_warmblood1 = 0.3456 |
| const double | EM\_warmblood2 = 5.9856 |
| const double | ExtraBroodHeat = 2.4 / KcalPerGInsect |
| double | FemaleNestAcceptScore |
| static int | FoodTripsPerDay |
| Landscape \* | g\_land |
| CfgInt | g\_map\_maxpolyref |
| boost::variate\_generator  < base\_generator\_type   &, boost::uniform\_real<> > | g\_rand\_uni |
| int | g\_stopdate = July+14 |
| double | HeightScore [111] |
| const int | HomeRangeDiameter = 280 |
| const int | HomeRangeDiameterDiv20 = 14 |
| int | HQualityBareEarth = 0 |
| int | HQualityGood = 30 |
| int | HQualityHedge = -1050 |
| int | HQualityHedgeScrub = 0 |
| int | HQualityMetalRoad = -10 |
| int | HQualityNeutral = 0 |
| int | HQualityOpenTallVeg = 16 |
| int | HQualityTall = 0 |
| int | HQualityTall2 = -1000 |
| int | HQualityTallVeg = 1 |
| int | HQualityTrack = 10 |
| int | HQualityVeg30cm = 11 |
| int | HQualityWater = 0 |
| double | IsTramline |
| int | JuvenileReturnMort |
| const double | KcalPerGInsect = 5.0 |
| const double | KcalPerGInsect\_inv = 1.0 / KcalPerGInsect |
| const double | KcalPerGInsect\_kg\_inv = KcalPerGInsect\_inv/1000.0 |
| static const unsigned int | MapDumpColours [34] |
| double | MaxFeedRain |
| static double | MD\_Hatch |
| static double | MD\_Threshold |
| static double | MeanExtractionRatePerMinute |
| static double | MeanHatchingWeight |
| static double | NestLeavingWeight |
| static int | NestlingMortProb |
| double | NestPlacementMinQual = 15 |
|  | The minimum quality for nest placement. |
| double | NotTramline |
| int | PatchyPremium = 10 |
| static int | PreFledgeMortProb |
| double | RainHindrance [21] |
| const AnsiString | SimulationName = "Skylark" |
| FILE \* | StriglingMort |
| double | TempHindrance [31] |
| double | TerrHeterogeneity [1000] |
| const int | terrnos [6] |
| const int | terrsize [6] |
| int | TramlinePremium = 5 |
| double | VegHindranceD [111] |
| double | VegHindranceH [111] |
| const double | VegQuality [31] |
| const double | VeryHighDensityVeg = 2200.0 |
| double | XFNestAcceptScore |

---

## Macro Definition Documentation

#define \_\_CJTDebug\_7

#define SKOPTIMALHABITATSCORE   50

Referenced by SkTerritories::PrePoly2Qual(), and SkTerritories::PrePolyNQual().

---

## Function Documentation

|  |  |  |  |
| --- | --- | --- | --- |
| bool CompareDist | ( | Point | *i*, |
|  |  | Point | *j* |
|  | ) |  |  |

References Point::m\_y.

Referenced by SkTerritories::GetTerritoriesByDistance().

{ return (i.m\_y > j.m\_y); }

---

## Variable Documentation

int Breed\_Res\_Thresh1

static

Referenced by Skylark\_Population\_Manager::LoadParameters().

const double Breed\_Res\_Thresh2 = Breed\_Res\_Thresh1 \* 4

const double Breed\_Temp\_Thresh = 5.0

Referenced by Skylark\_Female::st\_BuildingUpResources().

const double CE\_nest[15]

**Initial value:**

{

0, 0, 0.91082709, 0.827867467, 0.752463941, 0.683928293, 0.621634983, 0.565015451, 0.513552918, 0.466777677, 0.424262801,

0.38562025, 0.350497326, 0.318573455, 0.289557263

}

Referenced by Skylark\_Nestling::On\_FoodSupply().

CfgInt cfg\_adultreturnmort

Immigration mortality for juveniles.

CfgInt cfg\_Breed\_Res\_Thresh1

CfgBool cfg\_CIPEGridOutput\_used

Referenced by Population\_Manager::Run().

CfgInt cfg\_ClutchMortProb

CfgFloat cfg\_ConversionEffReduc

CfgFloat cfg\_Cooling\_Rate\_Eggs

Maximum immigration mortality for females.

Minimum immigration mortality for females Maximum immigration mortality for males Minimum immigration mortality for males

CfgFloat cfg\_densityconstant\_a

CfgFloat cfg\_densityconstant\_b

CfgInt cfg\_densityconstant\_c

CfgFloat cfg\_EggTemp

CfgFloat cfg\_EM\_Nestling\_a

CfgFloat cfg\_EM\_Nestling\_b

CfgInt cfg\_fecundity\_reduc

CfgInt cfg\_fecundity\_reduc\_chance

CfgFloat cfg\_FemaleMinTerritoryAcceptScore

CfgInt cfg\_FoodTripsPerDay

CfgFloat cfg\_heightconstant\_a

CfgFloat cfg\_heightconstant\_b

CfgInt cfg\_heightconstant\_c

CfgFloat cfg\_hindconstantD\_b

CfgFloat cfg\_hindconstantH\_b

CfgInt cfg\_HQualityBareEarth

CfgInt cfg\_HQualityHedge

CfgInt cfg\_HQualityHedgeScrub

CfgInt cfg\_HQualityMetalRoad

CfgInt cfg\_HQualityNeutral

CfgInt cfg\_HQualityOpenTallVeg

CfgInt cfg\_HQualityTall

CfgInt cfg\_HQualityTall2

CfgInt cfg\_HQualityTallVeg

CfgInt cfg\_HQualityTrack

CfgInt cfg\_HQualityVeg30cm

CfgInt cfg\_HQualityWater

CfgInt cfg\_insecticide\_direct\_mortE

CfgInt cfg\_insecticide\_direct\_mortF

CfgInt cfg\_insecticide\_direct\_mortM

CfgInt cfg\_insecticide\_direct\_mortN

CfgInt cfg\_insecticide\_direct\_mortP

CfgInt cfg\_juvreturnmort

Immigration mortality for juveniles.

CfgFloat cfg\_MaleSplitScale

CfgFloat cfg\_maxfeedrain

CfgFloat cfg\_MD\_Threshold

CfgFloat cfg\_MeanExtractionRatePerMinute

CfgFloat cfg\_MeanHatchingWeight

CfgFloat cfg\_MinDaysToHatch

The time taken to egg hatch under optimal conditions.

CfgInt cfg\_NestLeavingChance

CfgFloat cfg\_NestLeavingWeight

CfgInt cfg\_NestlingMortProb

CfgFloat cfg\_NestPlacementMinQual

CfgInt cfg\_PatchyPremium

CfgFloat cfg\_PEmax

CfgInt cfg\_pm\_eventfrequency

CfgInt cfg\_pm\_eventsize

CfgInt cfg\_PreFledgeMortProb

CfgInt cfg\_rainhindpow

CfgBool cfg\_ReallyBigOutput\_used

Referenced by Population\_Manager::Run(), and Population\_Manager::~Population\_Manager().

CfgBool cfg\_ReallyBigOutputMonthly\_used

CfgBool cfg\_RipleysOutput\_used

Referenced by Population\_Manager::Run(), and Population\_Manager::~Population\_Manager().

CfgFloat cfg\_SK\_hindrance\_scale

CfgFloat cfg\_sk\_triplength

CfgFloat cfg\_SkScrapesPremiumII

CfgInt cfg\_SkStartNos

The number of skylarks that start in the simulation.

Parameters under control of the config file \*\*\*\*\*\*\*\*\*\*\*\*\*\*\*\*\*\*\*

CfgInt cfg\_SkTramlinesPremium

CfgFloat cfg\_Skylark\_female\_Biodegredation

The proportion of pesticide remaining from one day to the next for females.

CfgFloat cfg\_Skylark\_female\_NOEL

Can be used to trigger a response to pesticides for the females.

CfgFloat cfg\_Skylark\_male\_Biodegredation

The proportion of pesticide remaining from one day to the next for males.

CfgFloat cfg\_Skylark\_male\_NOEL

Can be used to trigger a response to pesticides for the males.

CfgFloat cfg\_Skylark\_nestling\_Biodegredation

The proportion of pesticide accumulated from one day to the next.

CfgFloat cfg\_Skylark\_nestling\_NOEL

Can be used to trigger a response to pesticides for the nestlings.

CfgFloat cfg\_Skylark\_prefledegling\_Biodegredation

The proportion of pesticide remaining from one day to the next for prefledglings.

CfgFloat cfg\_Skylark\_prefledegling\_NOEL

Used for determining the pesticide response for prefledglings.

CfgInt cfg\_strigling\_clutch

CfgInt cfg\_strigling\_nestling

CfgInt cfg\_strigling\_preflg

CfgInt cfg\_temphindpow

CfgFloat cfg\_tramline\_foraging

int ClutchMortProb

static

Referenced by Skylark\_Population\_Manager::LoadParameters(), and Skylark\_Clutch::st\_Developing().

double Cooling\_Rate\_Eggs

static

Referenced by Skylark\_Population\_Manager::LoadParameters(), and Skylark\_Clutch::st\_Developing().

double DensityScore[111]

Referenced by Skylark\_Population\_Manager::LoadParameters(), and SkTerritories::PrePolyNQual().

double EggTemp

static

Referenced by Skylark\_Population\_Manager::LoadParameters(), and Skylark\_Clutch::st\_Developing().

const double EM\_coldblood1 = 0.4257

const double EM\_coldblood2 = 0.482

const double EM\_nest\_T[14]

**Initial value:**

{

0.0, 0.04958283, 0.077473171, 0.117759221, 0.148748489, 0.179737758, 0.322288393, 0.362574442, 0.38426693, 0.409058345,

0.436948687, 0.399761565, 0.418355126, 0.440047614,

}

Referenced by Skylark\_Nestling::st\_Developing().

const double EM\_warmblood1 = 0.3456

const double EM\_warmblood2 = 5.9856

const double ExtraBroodHeat = 2.4 / KcalPerGInsect

double FemaleNestAcceptScore

Referenced by Skylark\_Population\_Manager::LoadParameters().

int FoodTripsPerDay

static

Referenced by Skylark\_Female::FeedYoung(), Skylark\_Population\_Manager::LoadParameters(), and Skylark\_Male::st\_CaringForYoung().

Landscape\* g\_land

CfgInt g\_map\_maxpolyref

Referenced by Landscape::Landscape(), and Landscape::ReadPolys().

boost::variate\_generator<base\_generator\_type&, boost::uniform\_real<> > g\_rand\_uni

int g\_stopdate = July+14

Referenced by Skylark\_Female::OnMateDying(), Skylark\_Male::OnMateDying(), Skylark\_Female::OnMateHomeless(), Skylark\_Male::OnMateLeaving(), Skylark\_Male::st\_AttractingAMate(), Skylark\_Female::st\_BuildingUpResources(), Skylark\_Female::st\_CaringForYoung(), Skylark\_Female::st\_Emigrating(), Skylark\_Male::st\_Emigrating(), Skylark\_Female::st\_Floating(), Skylark\_Male::st\_Floating(), Skylark\_Female::st\_PreparingForBreeding(), and Skylark\_Female::st\_StartingNewBrood().

double HeightScore[111]

Referenced by Skylark\_Population\_Manager::LoadParameters(), and SkTerritories::PrePolyNQual().

const int HomeRangeDiameter = 280

const int HomeRangeDiameterDiv20 = 14

Referenced by SkTerritories::PreFillTerrPolyLists().

int HQualityBareEarth = 0

Referenced by Skylark\_Population\_Manager::LoadParameters(), SkTerritories::PrePoly2Qual(), and SkTerritories::PrePolyNQual().

int HQualityGood = 30

int HQualityHedge = -1050

Referenced by Skylark\_Population\_Manager::LoadParameters(), SkTerritories::PrePoly2Qual(), and SkTerritories::PrePolyNQual().

int HQualityHedgeScrub = 0

Referenced by Skylark\_Population\_Manager::LoadParameters(), SkTerritories::PrePoly2Qual(), and SkTerritories::PrePolyNQual().

int HQualityMetalRoad = -10

Referenced by Skylark\_Population\_Manager::LoadParameters(), SkTerritories::PrePoly2Qual(), and SkTerritories::PrePolyNQual().

int HQualityNeutral = 0

Referenced by Skylark\_Population\_Manager::LoadParameters().

int HQualityOpenTallVeg = 16

Referenced by Skylark\_Population\_Manager::LoadParameters().

int HQualityTall = 0

Referenced by Skylark\_Population\_Manager::LoadParameters(), SkTerritories::PrePoly2Qual(), and SkTerritories::PrePolyNQual().

int HQualityTall2 = -1000

Referenced by Skylark\_Population\_Manager::LoadParameters(), SkTerritories::PrePoly2Qual(), and SkTerritories::PrePolyNQual().

int HQualityTallVeg = 1

Referenced by Skylark\_Population\_Manager::LoadParameters(), SkTerritories::PrePoly2Qual(), and SkTerritories::PrePolyNQual().

int HQualityTrack = 10

Referenced by Skylark\_Population\_Manager::LoadParameters().

int HQualityVeg30cm = 11

Referenced by Skylark\_Population\_Manager::LoadParameters().

int HQualityWater = 0

Referenced by Skylark\_Population\_Manager::LoadParameters(), SkTerritories::PrePoly2Qual(), and SkTerritories::PrePolyNQual().

double IsTramline

Referenced by Skylark\_Population\_Manager::LoadParameters().

int JuvenileReturnMort

Referenced by Skylark\_Male::GetMigrationMortality(), and Skylark\_Population\_Manager::Skylark\_Population\_Manager().

const double KcalPerGInsect = 5.0

const double KcalPerGInsect\_inv = 1.0 / KcalPerGInsect

const double KcalPerGInsect\_kg\_inv = KcalPerGInsect\_inv/1000.0

Referenced by Skylark\_Male::GetFood().

const unsigned int MapDumpColours[34]

static

Referenced by SkTerritories::DumpMapGraphics().

double MaxFeedRain

Referenced by Skylark\_Female::FeedYoung(), Skylark\_Population\_Manager::LoadParameters(), Skylark\_Male::st\_CaringForYoung(), and Skylark\_Female::st\_Incubating().

double MD\_Hatch

static

Referenced by Skylark\_Population\_Manager::LoadParameters(), and Skylark\_Clutch::st\_Developing().

double MD\_Threshold

static

Referenced by Skylark\_Population\_Manager::LoadParameters(), and Skylark\_Clutch::st\_Developing().

double MeanExtractionRatePerMinute

static

Referenced by Skylark\_Population\_Manager::LoadParameters(), and Skylark\_Adult::Skylark\_Adult().

double MeanHatchingWeight

static

Referenced by Skylark\_Population\_Manager::LoadParameters(), and Skylark\_Nestling::Skylark\_Nestling().

double NestLeavingWeight

static

Referenced by Skylark\_Population\_Manager::LoadParameters(), and Skylark\_Nestling::st\_Developing().

int NestlingMortProb

static

Referenced by Skylark\_Population\_Manager::LoadParameters(), and Skylark\_Nestling::st\_Developing().

double NestPlacementMinQual = 15

The minimum quality for nest placement.

Referenced by Skylark\_Population\_Manager::LoadParameters().

double NotTramline

Referenced by Skylark\_Population\_Manager::LoadParameters().

int PatchyPremium = 10

Referenced by Skylark\_Population\_Manager::LoadParameters(), SkTerritories::PrePoly2Qual(), and SkTerritories::PrePolyNQual().

int PreFledgeMortProb

static

Referenced by Skylark\_Population\_Manager::LoadParameters(), and Skylark\_PreFledgeling::st\_Developing().

double RainHindrance[21]

Referenced by Skylark\_Adult::GetWeatherHindrance(), and Skylark\_Population\_Manager::LoadParameters().

const AnsiString SimulationName = "Skylark"

FILE\* StriglingMort

double TempHindrance[31]

Referenced by Skylark\_Adult::GetWeatherHindrance(), and Skylark\_Population\_Manager::LoadParameters().

double TerrHeterogeneity[1000]

const int terrnos[6]

**Initial value:**

{

0, 1, 2, 4, 5, 10

}

const int terrsize[6]

**Initial value:**

{

0, 500, 250, 125, 100, 50

}

int TramlinePremium = 5

double VegHindranceD[111]

Referenced by Skylark\_Adult::GetVegHindrance(), and Skylark\_Population\_Manager::LoadParameters().

double VegHindranceH[111]

Referenced by Skylark\_Adult::GetVegHindrance(), and Skylark\_Population\_Manager::LoadParameters().

const double VegQuality[31]

**Initial value:**

{

30, 30, 29.6, 27.2, 24.8, 23, 21.2, 19.6, 18.2, 17, 15.8, 15, 14.2, 13.6, 13, 12.6, 12.2, 12, 11.8, 11.6, 11.6, 11.6, 11.6,

11.6, 11.6, 11.6, 11.4, 11.4, 11.2, 11.2, 10.8

}

const double VeryHighDensityVeg = 2200.0

double XFNestAcceptScore


- CJT
- MSVC
- ALMaSS Working Source
- Skylark
- skylarks\_all.cpp
- Generated on Thu Jan 10 2013 13:15:35 for ALMaSS Skylark ODdox by
   1.8.1.1
